# Ultrafast and Energy-saving Synthesis of Nitrogen and Chlorine Co-doped Carbon Nanodots via Neutralization Heat for Selective Detection of Cr(VI) in Aqueous Phase

**DOI:** 10.3390/s18103416

**Published:** 2018-10-11

**Authors:** Qin Hu, Tao Li, Lu Gao, Xiaojuan Gong, Shengqi Rao, Weiming Fang, Ruixia Gu, Zhenquan Yang

**Affiliations:** 1 College of Food Science and Engineering, Yangzhou University, Jiangsu 225001, China; qinhu1998@outlook.com (Q.H.); gaolu@yzu.edu.cn (L.G.); sqrao@yzu.edu.cn (S.R.); wmfang@yzu.edu.cn (W.F.); rxgu@yzu.edu.cn (R.G.); 2 Jiangsu Key Laboratory of Dairy Biotechnology and Safety Control, Yangzhou University, Jiangsu 225001, China; 3 Department of Agronomy, Yangzhou University, Jiangsu 225001, China; taoli@yzu.edu.cn; 4 Institute of Environmental Science, and School of Chemistry and Chemical Engineering, Shanxi University, Taiyuan 030006, China; gxj1124@sxu.edu.cn

**Keywords:** carbon nanodots, Cr(VI) detection, fluorescent nanosensor, water samples

## Abstract

In this work, it is presented for the first time that nitrogen and chlorine co-doped carbon nanodots (*N*,*Cl*-CDs) were synthesized by simply mixing glucose, concentrated hydrochloric acid (HCl), and 1,2-ethylenediamine (EDA). No external heat was employed; the neutralization reaction served as the heat source. The glucose served as the carbon source while EDA and HCl were the N and Cl dopants, respectively. The fluorescence of *N*,*Cl*-CDs was adequately quenched by hexavalent chromium Cr(VI) based on a combination of dynamic quenching and inner filter effect (IFE). Accordingly, an efficient *N*,*Cl*-CDs-based fluorescence probe was established for sensitive and selective detection of Cr(VI). The proposed fluorescence sensor provides a linear recognition range for Cr(VI) determination from 3 to 40 µM with a limit of detection (LOD) of 0.28 µM (14.6 µg/L). The proposed fluorescence method was successfully utilized to detect Cr(VI) in different water samples with satisfactory results. The spike recoveries vary from 97.01% to 103.89% with relative standard deviations (RSDs) of less than 0.82%. This work highlights the development of a simple, ultrafast, and energy-saving one-step synthetic route to fabricate *N*,*Cl*-CDs for highly selective and sensitive detection of Cr(VI) in real water samples. It is anticipated that the proposed fluorescence method could be further explored and widely used for Cr(VI) detection in the environmental industry.

## 1. Introduction

Fluorescent carbon nanodots (CDs) with typical sizes below 10 nm have received considerable attention due to their excellent optical features such as upconversion photoluminescence (PL), tunable excitation/emission, high photostability, low cytotoxicity, and excellent biocompatibility [1]. The PL property of C-dots has generated numerous publications on various applications such as bioimaging [2,3] and nanosensors [4,5]. So far, numerous accounts of efforts have been devoted to enhancing the PL performance of CDs [6,7,8] and extending their applications in analysis and sensing. Heteroatoms doping is considered to be the most useful approach to improve fluorescence properties of CDs [9]. Nitrogen (N) doping was the first approach employed to improve the PL performance of CDs. N doping could introduce defect sites and provide potential active sites to CDs, leading to substantial fluorescence enhancement of CDs; however, some reported N-CDs are restricted from low aqueous dispersity or weak resistance to metal ion interference, acid or base [10,11,12]. Fortunately, doping other heteroatoms in combination with N was found to be useful in overcoming these shortcomings [12,13,14]. Extensive reports have shown that combining sulfur (S), phosphorus (P), boron (B), or fluorine (F) with N is efficient in increasing aqueous dispersity and promoting selectivity and sensitivity. For example, Feng et al. synthesized N- and S-co-doped CDs (NSCDs) by microwave-assisted pyrolysis of vitamin C and thiourea and utilized it as a fluorescent sensor for Fe^3+^ detection [15]. Wang et al. reported the fabrication of N-, S-, and P-co-doped CDs (N/S/P-CDs) by hydrothermal treatment of cucumber juice for Hg^2+^ detection [16]. Liu et al. produced an efficient fluorescent sensor based on B-, N-, S-co-doped carbon dots (BNS-CDs) by hydrothermal treatment of 2,5-diaminobenzenesulfonic acid and 4-aminophenylboronic acid hydrochloride for Fe^3+^ detection [17]. Zuo et al. introduced F into N-doped CDs by solvothermal process using aromatic F bearing moiety as the F source and applied it for selective detection of Ag^+^ [11]. Although CDs doped with different heteroatoms, including N, S, P, B, F, and their combinations, have been reported, the fabrication of efficient CDs is still highly demanded due to the intense interest in various CD-based applications and therefore, it is strongly desirable to explore other heteroatom doping systems. Some cation ions such as Al^3+^ [18] and Co^2+^ [19] were reported to be useful in improving the fluorescence property of CDs; however, the toxicity of the metal-ions-doped CDs is still unknown, and the applications of the metal-ions-doped CDs are rarely reported. Recently, a new type of N- and chlorine (Cl)-co-doped CDs (*N*,*Cl*-CDs) with high aqueous dispersity was reported by hydrothermal treatment of D-Glucosamine hydrochloride and was found to be efficient in selectively detecting Fe^3+^ [20]. However, to the best of our knowledge, this is the only work that has been contributed to fabricating *N*,*Cl*-CDs, and the as-synthesized *N*,*Cl*-CDs have not been explored for other applications yet.

Several heavy metals are known to be hazardous to the environment and living organisms. Among the known hazardous metal ions, hexavalent chromium Cr(VI) is considered as one of the most dangerous ones in comparison with the other valence states of chromium, such as Cr(0) and Cr(III), due to its high toxicity and carcinogenic property [21]. Exposure to Cr(VI) will cause damage to kidney, liver, and other organs, leading to hemolysis, liver failure, and even cancer [22]. As such, the development of an accurate and convenient detection method for Cr(VI) is of significant importance. In the past decades, various detection methods have been developed for the recognition of Cr(VI), including atomic absorption spectroscopy [23], liquid/gas chromatography [24], spectrophotometry [25], and inductively coupled plasma spectrometry [26]. However, these methods are not able to distinguish Cr(VI) from Cr(III) and can only measure the total amount of chromium, and they generally require time-consuming sample pretreatments, expensive and large instrumentation, and complex operations. Recently, CD-based sensors [22,27] have attracted much attention in Cr(VI) detection due to their advantages in low cost, favorable selectivity, and ease of miniaturization. So far, a bunch of CDs have been developed for Cr(VI) recognition with high selectivity and sensitivity; however, most of the preparation procedures for CDs require external heat and long heating time to achieve the carbonization of carbon source [28,29,30,31], and some of the reported methods were only performed in the preliminary test stage with no real sample application employed [28,29]. As such, the development of a simple, fast, and energy-efficient synthetic route to produce highly selective and sensitive CDs with the capability for Cr(VI) detection in real samples is highly desirable. 

In this work, a simple, ultrafast, and energy-saving one-step approach was presented to prepare *N*,*Cl*-CDs for Cr(VI) recognition with neutralization reaction as the heat source, glucose as carbon source, and the concentrated hydrochloric acid (HCl) and 1,2-ethylenediamine (EDA) as Cl and N dopants, respectively. No external heat source was employed; the dehydration and carbonization of glucose was achieved within 2 min via the neutralization heat. The obtained *N*,*Cl*-CDs solution exhibited long-term colloidal stability in aqueous phase at ambient conditions for two months. The fluorescence emission of the as-synthesized *N*,*Cl*-CDs could be strongly quenched by Cr(VI) via a synergistic effect of dynamic quenching and inner filter effect (IFE). The analytical performance of the proposed fluorescent method was comprehensively studied by evaluating its selectivity and sensitivity in measuring Cr(VI). The proposed fluorescent method was successfully applied for the analysis of Cr(VI) in real water samples. As far as we are aware, this is the simplest and fastest method to fabricate *N*,*Cl*-CDs which has the capability for Cr(VI) detection in real samples, which certainly adds to the database of the selective and sensitive detection of Cr(VI) by using fluorescent probes.

## 2. Materials and Methods

### 2.1. Materials

Glucose, concentrated hydrochloric acid (HCl), and 1,2-ethylenediamine (EDA) were bought from Aldrich (Milwaukee, WI, USA). The quinine sulfate was purchased from Shanghai Macklin Biochemical Technology Co., Ltd. (Shanghai, China). The Dulbecco’s modified Eagle’s medium (DMEM), dimethyl sulfoxide (DMSO), 3-(4,5-dimethylthiazol-2-yl)-2,5-diphenyltetrazolium bromide (MTT), and fetal bovine serum (FBS) were bought from Solarbio (Beijing, China). The other reagents used in this work were obtained from Sinopharm Chemical Reagent Co., Ltd. (Shanghai, China). The copper grids with the ultrathin carbon films were bought from Ted Pella (Redding, CA, USA). The ultrapure water used throughout this work was produced by a Milli-Q Plus water purification system (Bedford, MA, USA) with a resistivity higher than 18.25 MΩ·cm. All the reagents were used as received without further purification unless noted otherwise. 

### 2.2. Synthesis of Nitrogen/Chlorine Dual-Doped Carbon Nanodots (N,Cl-CDs)

In a typical synthetic procedure, 4.0 mL of concentrated HCl and 6.0 mL of EDA were quickly mixed with 0.4 g of glucose in a 50 mL glass beaker. No stirring was employed and the reaction mixture became foamy under the acid–base neutralization’s spontaneous heat. When the reaction was over, the reaction mixture was naturally cooled down to room temperature. The resultant reaction product was purified by a Spectrum Laboratories (Rancho Dominguez, CA, USA) dialysis membrane tube with a molecular weight cut-off 500–1000 Da against 1L ultrapure water with stirring and recharging with fresh ultrapure water every 24 h over a course of 3 days. Finally, the *N*,*Cl*-CDs solution in dark brown color was filtered and freeze-dried to get dry *N*,*Cl*-CDs product.

### 2.3. Characterization of the As-Synthesized N,Cl-CDs

The transmission electron microscopic (TEM) image was acquired on a JEM-2100 transmission electron microscope (JEOL, Tokyo, Japan). The elemental analysis of C, H, and N content was performed on an Analysensysteme vario EL cube organic elemental analyser (Elementar, Hanau, Germany) while the elemental analysis of Cl content was carried out on a multi EA^®^ 4000 Elemental Analyzer (Analytik Jena AG, Jena, Germany). The Fourier transform infrared spectrum (FTIR) was performed on a Tensor II FTIR spectrometer (Bruker, Bremen, Germany). The UV-vis spectra were recorded on a UV-2550 absorption spectrophotometer (Shimadzu Co., Ltd., Tokyo, Japan) and the photoluminescence (PL) spectra were carried out on a RF-5301PC fluorescence spectrophotometer (Shimadzu Co., Ltd., Tokyo, Japan). Fluorescence lifetime assays were carried out on a FLS-980 fluorescence spectrophotometer (Edinburgh Instruments Ltd., Livingston, England).

### 2.4. Determination of Cr(VI) 

The *N*,*Cl*-CDs, metal ions, and anion ions used for the experiments were dissolved in purified water. To study the selectivity of *N*,*Cl*-CDs towards Cr(VI) sensing, the stock solutions of 18 kinds of ions (such as K^+^, Na^+^, Pb^2+^, Ag^+^, Fe^3+^, Al^3+^, Fe^2+^, Ca^2+^, Mn^2+^, Mg^2+^, Cd^2+^, Ni^2+^, Co^2+^, Cu^2+^, Ba^2+^, Zn^2+^, Cr^3+^, and Cr(VI)) and 15 kinds of anion ions (F^−^, Cl^−^, Br^−^, I^−^, NO_3_^−^, NO_2_^−^, H_2_PO_4_^−^, SCN^−^, SO_4_^2−^, CO_3_^2−^, S_2_O_3_^2−^, HPO_4_^2−^, SO_3_^2−^, C_2_O_4_^2−^, and Cr_2_O_7_^2−^) were prepared in a concentration of 0.15 mol/L, respectively. Then, each 10 µL of the above metal ions and anion ions stock solutions (0.15 mol/L) was mixed with 3.0 mL of the *N*,*Cl*-CDs solution (1.25 mg/mL), and the PL intensities were recorded. A mixture of 10 µL of purified water and 3.0 mL of the *N*,*Cl*-CDs solution (1.25 mg/mL) was used as a control sample. The fluorescence intensities were recorded at an excitation/emission wavelength (λ_ex_/λ_em_) of 380/467 nm.

To investigate the interferences of the possible interfering metal and anion ions towards Cr(VI) sensing, each 10 µL of K^+^, Na^+^, Pb^2+^, Ag^+^, Fe^3+^, Al^3+^, Fe^2+^, Ca^2+^, Mn^2+^, Mg^2+^, Cd^2+^, Ni^2+^, Co^2+^, Cu^2+^, Ba^2+^, Zn^2+^, Cr^3+^, F^−^, Cl^−^, Br^−^, I^−^, NO_3_^−^, NO_2_^−^, H_2_PO_4_^−^, SCN^−^, SO_4_^2−^, CO_3_^2−^, S_2_O_3_^2−^, HPO_4_^2−^, SO_3_^2−^, and C_2_O_4_^2−^ stock solutions (0.15 mol/L) was mixed with 3.0 mL *N*,*Cl*-CDs (1.25 mg/mL), respectively, and then 10 µL of the stock solution of Cr(VI) (0.15 mol/L) was introduced. A mixture of 0.1 mL of purified water, 3.0 mL *N*,*Cl*-CDs (1.25 mg/mL), and 10 µL of the stock solution of Cr(VI) (0.15 mol/L) was used as a control sample. The fluorescence intensities were measured at a λ_ex_/λ_em_ of 380/467 nm.

To determine the linearity of *N*,*Cl*-CDs towards Cr(VI) sensing, different volumes of 0.01 mol/L Cr(VI) were incrementally titrated into 3.0 mL *N*,*Cl*-CDs (1.25 mg/mL). The fluorescence intensities were recorded at a λ_ex_/λ_em_ of 380/467 nm.

### 2.5. Real Sample Analysis

In analysis of real water samples, tap water from our lab in Yangzijin Campus of Yang University, rain water collected during raining days at Yangzhou City, and river water A and B from local rivers at Yangzhou City were sampled. For the pretreatment, the tap water sample was used as received while the rain water and the river water samples were filtered through 0.45 µm, 13 mm id cellulose acetate syringe filters (Tianjin Jinteng Experiment Equipment Co., Ltd., Tianjin, China) before analysis. A standard addition method was employed to measure the Cr(VI) content in water samples. In detail, 3 mL of the *N*,*Cl*-CDs solutions (1.25 mg/mL) were introduced separately into 5.0 mL round bottom centrifuge tubes, and then 10 µL of water samples and 10 µL of Cr(VI) solution at four concentration levels were added, making the final concentrations of the spiked Cr(VI) at 5, 10, 20 and 30 uM. The fluorescence intensities were recorded at a λ_ex_/λ_em_ of 380/467 nm.

### 2.6. MTT Assay

The cytotoxicity of *N*,*Cl*-CDs was assessed using human cervical cancer SiHa cells. The SiHa cells were cultured in 200 mL of fresh DMEM medium containing 10% FBS at 37 °C in 5.0% CO_2_ and 95% atmospheric environment for 3 h in a Costar^®^ 96-well cell plate. Then, serial dilutions of the *N*,*Cl*-CDs solutions were introduced into the wells, making the final concentration levels of *N*,*Cl*-CDs in the medium at 50, 100, 200, 400 and 800 mg/mL. The wells that contained the cells without the treatment by *N*,*Cl*-CDs were used as negative control. After 48 h, 20 mL of the MTT reagent (5.0 mg/mL) was introduced. After further incubation for 4 h, the medium inside each well was removed and 150 mL of DMSO was introduced to mix. The optical density (OD) of the mixture was recorded using a SunRise microplate reader (Tecan Austria GmbH, Grödig, Austria) at a λ_ex_ of 490 nm. At least six independent experiments were performed to acquire accurate data. The following equation was used to calculate the cell viability:

Cell viability (%) = (OD_Treated_/OD_Control_) × 100% (1)

where OD_Treated_ and OD_Control_ are the OD obtained in the absence and in the presence of *N*,*Cl*-CDs, respectively.

## 3. Results and Discussion

### 3.1. Characterization of N,Cl-CDs

**Figure 1:**
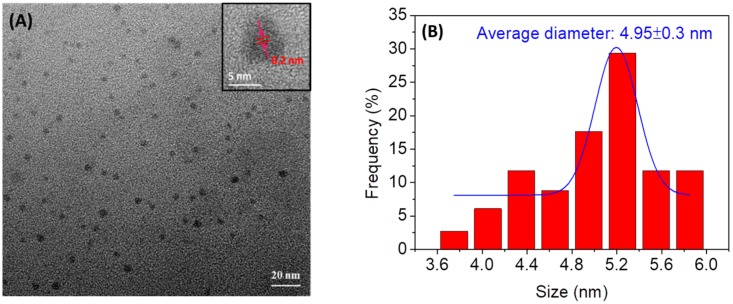
(**A**) TEM image and (**B**) particle size distribution of the as-fabricated *N*,*Cl*-CDs. The inset in the top right of Figure 1A is a magnified TEM image of an *N*,*Cl*-CDs particle.

The morphology and size of *N*,*Cl*-CDs were determined by TEM. A drop of aqueous sample of *N*,*Cl*-CDs was spotted onto the ultrathin carbon film coated on a copper grid. As depicted in Figure 1A, the *N*,*Cl*-CDs are mostly of spherical morphology and evenly dispersed on the TEM image. The absence of any agglomerate of *N*,*Cl*-CDs shows the good aqueous dispersity of *N*,*Cl*-CDs. The corresponding histogram obtained by counting randomly 80 particles from various spots of the TEM image is depicted in Figure 1B. It is revealed that the *N*,*Cl*-CDs had a size range of 3.7–5.8 nm with an average diameter of 4.95 ± 0.3 nm. In addition, a magnified TEM image of *N*,*Cl*-CDs is displayed in the inset of Figure 1A. The *N*,*Cl*-CDs were revealed to have a graphitic structure with the lattice spacing of ca. 0.20 nm which is consistent with the (100) facet of graphitic structure [32,33].

The doping of heteroatoms into *N*,*Cl*-CDs was initially probed by elemental analysis. As shown in Table S1, the *N*,*Cl*-CDs were comprised of C 25.88, H 9.40, N 25.69, Cl 23.29 wt%, and O (calculated) 15.74 wt%, indicating the dual-doping of nitrogen and chlorine into CDs. The empirical formula for the *N*,*Cl*-CDs is C_6_H_28_N_6_Cl_2_O_3_. To characterize the functionalities of *N*,*Cl*-CDs, the FTIR spectroscopy experiments were carried out. As shown in Figure S1, the *N*,*Cl*-CDs showed a sharp absorption peak at 3259 cm^−1^ and a broad absorption peak at 2996–3207 cm^−1^ which are ascribed to the N‒H stretching vibration and the O‒H stretching vibrations, respectively. The peak at 2772 cm^−1^ is attributed to the stretching vibration of C–H. Three sharp peaks at 1624, 1544, and 1355 cm^−1^ are corresponding to C=O stretching vibration, ‒CH_3_ umbrella bending, and C‒O stretching vibration, respectively. The peak at 823 cm^−1^ is attributed to C–Cl stretching vibration. All these observations indicate the presence of hydroxyl, carboxylic, amine, and alkyl chloride functionalities in *N*,*Cl*-CDs, which affords the *N*,*Cl*-CDs high hydrophilicity and good water dispersity.

**Figure 2:**
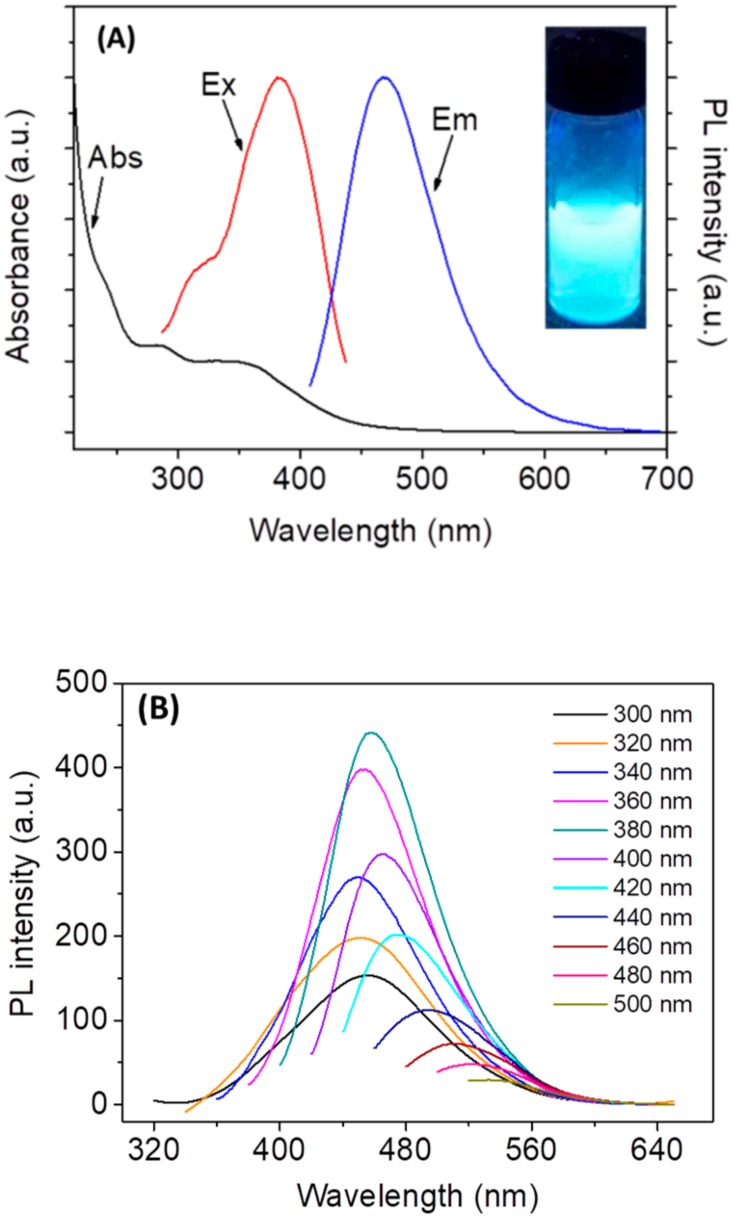
(**A**) The UV-vis absorption (black line), excitation (red line), and emission (blue line) spectra of *N*,*Cl*-CDs in aqueous solution (0.10 mg/mL). The inset shows the photograph of *N*,*Cl*-CDs aqueous solution (0.10 mg/mL) under UV light; (**B**) the PL spectra of *N*,*Cl*-CDs at different λ_ex_.

To study the optical properties of *N*,*Cl*-CDs, the UV-vis and PL spectra were acquired and are depicted in Figure 2. As shown by Figure 2A, the UV-vis spectrum of *N*,*Cl*-CDs (black line) showed two typical absorption peaks at 285 nm and 360 nm, with the former one originating from *n*→π* transition of C=O band and the latter one likely attributed to the excited defect surface states induced by N and Cl heteroatoms [28]. The excitation spectrum of *N*,*Cl*-CDs (red line) displayed two excitation peaks at 309 to 380 nm and the maximum excitation peak was 380 nm. The as-prepared *N*,*Cl*-CDs exhibited bright blue luminescence under UV light (Inset of Figure 2A). The PL spectrum of *N*,*Cl*-CDs (blue line) shows that the *N*,*Cl*-CDs displayed an emission peak at 467 nm when excited at 380 nm. As shown by Figure 2B, the *N*,*Cl*-CDs also revealed λ_ex_-dependent PL behavior, which is common with CDs [34,35,36] and is reported to arise from the presence of different particle sizes or various surface energy traps in *N*,*Cl*-CDs [37]. The quantum yield (QY) of *N*,*Cl*-CDs was determined to be 11.62% at a λ_ex_ of 380 nm using quinine sulfate as reference (Figure S2).

### 3.2. Optimization of the Experimental Conditions for Cr(VI) Detection

**Figure 3:**
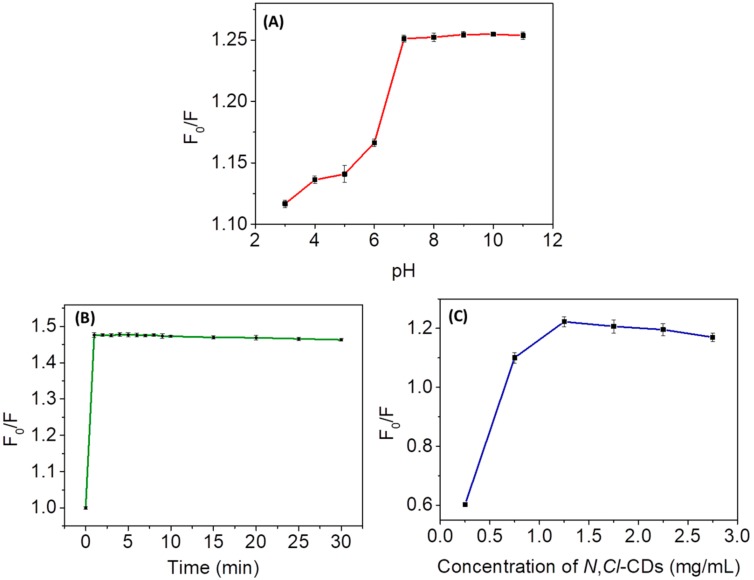
(**A**) Effect of pH on F_0_/F. 10 mM PBS solutions (pH 3‒11) were used for the experiments. The concentrations of *N*,*Cl*-CDs and Cr(VI) used were 1.25 mg/mL and 6 µM, respectively; (**B**) effect of reaction time on F_0_/F. The *N*,*Cl*-CDs and Cr(VI) were dissolved in ultrapure water, and their concentrations were 1.25 mg/mL and 6 µM, respectively; (**C**) effect of the concentration of *N*,*Cl*-CDs on F_0_/F. The *N*,*Cl*-CDs and Cr(VI) were dissolved in ultrapure water, and the concentration of Cr(VI) used was 6 µM.

For better analytical performances, the following parameters were optimized: (a) pH value, (b) reaction time and (c) concentration of *N*,*Cl*-CDs. The effect of pH on F_0_/F was firstly evaluated. F_0_ and F represent the fluorescence intensities of *N*,*Cl*-CDs (1.25 mg/mL) in the absence and in the presence of Cr(VI), respectively. The phosphate buffered saline (PBS) solutions at different pH values (3‒12) used here were prepared by mixing 10 mM Na_3_PO_4_, 10 mM Na_2_HPO_4_, and 10 mM NaH_2_PO_4_ in different proportions. As shown by Figure 3A, F_0_/F increased when pH increased from 3 to 7 and remained relatively stable in the pH range of 7–11. As such, the purified water (pH 7) was used as the solvent for the tests. The time response of *N*,*Cl*-CDs to Cr(VI) was also investigated to further optimize the analytical procedure. As shown by Figure 3B, there was no obvious change in F_0_/F after a reaction time of 1 min. As such, 1 min was selected as the optimal reaction time. Furthermore, the effect of *N*,*Cl*-CDs concentration (0.25‒2.75 mg/mL) on F_0_/F was investigated. As shown by Figure 3C, the maximum fluorescence quenching was observed when 1.25 mg/mL *N*,*Cl*-CDs was employed. Hence, 1.25 mg/mL was selected as the optimal concentration of *N*,*Cl*-CDs. In essence, pH 7, 1 min reaction time, and 1.25 mg/mL *N*,*Cl*-CDs were selected as the optimal parameters for subsequent analyses unless otherwise stated.

### 3.3. Selectivity of N,Cl-CDs to Various Ions

**Figure 4:**
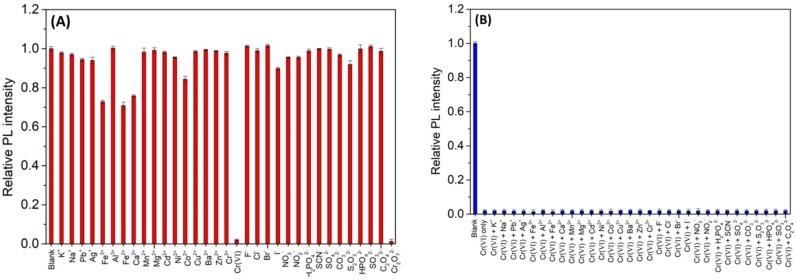
(**A**) The fluorescence intensity of *N*,*Cl*-CDs (1.25 mg/mL) after the addition of various metal ions and anion ions (0.5 mM); (**B**) the effects of coexisting ions (5 mM) on the fluorescence intensity of *N*,*Cl*-CDs (1.25 mg/mL)/Cr(VI) (0.5 mM).

The fluorescence response of *N*,*Cl*-CDs (1.25 mg/mL) to various metal ions and anion ions at a concentration of 0.5 mM were investigated and the results are shown in Figure 4A. It can be seen that among various ions, only Cr(VI) significantly quenched the fluorescence intensity of *N*,*Cl*-CDs while the other ions showed low or negligible influence on the fluorescence intensity of *N*,*Cl*-CDs, indicating that the *N*,*Cl*-CDs show high selectivity towards Cr(VI) over the other metal ions and anion ions.

### 3.4. Effects of Competition Ions

To monitor the effect of other interfering ions towards Cr(VI) detection, various metal and anion ions at a 10 times enhanced concentration (5 mM) were introduced into the *N*,*Cl*-CDs (1.25 mg/mL)/Cr(VI) (0.5 mM) system, respectively. As shown by Figure 4B, upon interaction with these ions, no pronounced changes were observed in the fluorescence intensity of the *N*,*Cl*-CDs/Cr(VI) system, indicating that the *N*,*Cl*-CDs have excellent anti-interference ability towards Cr(VI) detection.

In essence, the *N*,*Cl*-CDs show high selectivity to Cr(VI) over a wide scope of other possible interfering metal ions and anion ions and have excellent anti-interference ability to tolerate the interferences from high concentrations of the possible interfering metal ions and anion ions, affording *N*,*Cl*-CDs the capability for Cr(VI) detection in real samples.

### 3.5. Dependence of the Fluorescence of N,Cl-CDs on the Cr(VI) Concentration

**Figure 5:**
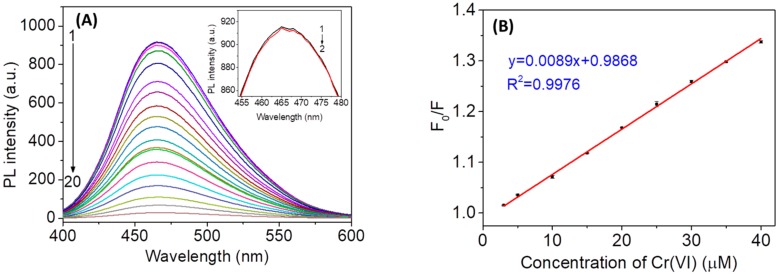
(**A**) Fluorescence quenching of *N*,*Cl*-CDs in the presence of different concentrations of Cr(VI). 1–20: 0, 0.93, 1.67, 4.17, 10, 20, 30, 40, 50, 60, 70, 80, 90, 100, 120, 150, 180, 230, 290, and 360 µM; the inset displays the magnified PL spectra of blank and *N*,*Cl*-CDs in the presence of 0.93 µM Cr(VI). (**B**) The SV plot of *N*,*Cl*-CDs at different concentrations of Cr(VI) in the linear range (3.00‒40.00 µM), where F_0_/F is the ratio of the fluorescence intensity of *N*,*Cl*-CDs (1.25 mg/mL) in the absence of Cr(VI) to that in the presence of Cr(VI).

In order to investigate the sensitivity and linear response range of *N*,*Cl*-CDs towards Cr(VI) detection, the fluorescence titrations were carried out. Figure 5A shows changes of the fluorescence intensity of *N*,*Cl*-CDs (1.25 mg/mL) after the addition of various concentrations of Cr(VI). The fluorescence intensity of *N*,*Cl*-CDs gradually decreased with the addition of Cr(VI) from 0.93 to 360 μM, indicating that Cr(VI) could effectively quench the fluorescence of the *N*,*Cl*-CDs. Figure 5B displays the plots of F_0_/F against the concentration of Cr(VI). The fluorescence intensity of *N*,*Cl*-CDs increased linearly with the concentration of Cr(VI) from 3 to 40 μM. The fluorescence quenching of *N*,*Cl*-CDs by Cr(VI) is fitted by Stern-Volmer (SV) equation:

F_0_/F = 1 + *K_SV_* [Q], (2)

where *K_SV_* is the SV quenching constant, [Q] is the concentration of Cr(VI), and F and F_0_ are the fluorescence intensities of *N*,*Cl*-CDs in the presence and in the absence of Cr(VI), respectively. The *K_SV_* is calculated to be 8.9× 10^3^ L/mol with a correlation coefficient *R^2^* of 0.9976. The limit of detection (LOD) is calculated as 3 times the standard deviation (SD) of twelve blank measurements (*n* = 12) divided by *K_SV_*, and the limit of quantification (LOQ) is defined as 3.3 times of LOD [28,38]. The LOD and LOQ of the proposed fluorescence method are calculated to be 0.28 µM (14.6 µg/L) and 0.93 µM (48.2 µg/L), respectively. The obtained LOD is lower than the acceptable concentration of Cr(VI) (50 µg/L) in drinking water regulated by the World Health Organization (WHO) [39]. This implies that the proposed *N*,*Cl*-CDs-based fluorescent nanosensor could be potentially used for the determination of Cr(VI) in environmental water.

### 3.6. Discussion of the Reaction Mechanism

**Figure 6:**
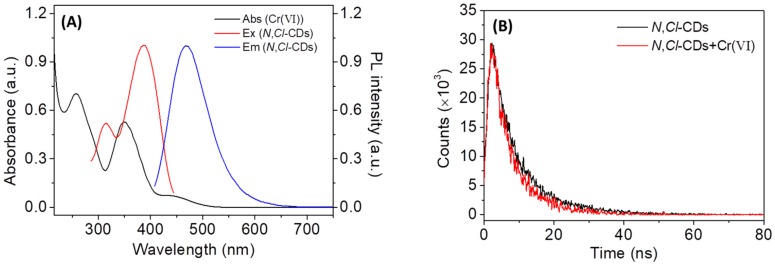
(**A**) The PL excitation (red line) and emission (blue line) spectra of *N*,*Cl*-CDs and UV-vis absorption spectrum of Cr(VI) (black line); (**B**) the fluorescence decay curves of *N*,*Cl*-CDs (1.25 mg/mL) in the absence and in the presence of 10 µM Cr(VI).

The IFE occurs if the absorption band of an absorber has overlap with the excitation and/or emission peak of a fluorophore to some extent [40,41]. Therefore, the designing of a high-efficiency, IFE-based fluorescent probe is interrelated with an appropriate absorber–fluorophore pair. To understand the fluorescence quenching mechanism, the fluorescence and UV-vis absorption spectroscopy were used to study the excitation and emission spectra of *N*,*Cl*-CDs and the absorption spectra of Cr(VI), respectively. As shown in Figure 6A, there are two peaks at 309 and 380 nm in the excitation spectrum of *N*,*Cl*-CDs (red line), and the maximum excitation peak is centered at 380 nm. Upon excitation at 380 nm, the *N*,*Cl*-CDs shows an emission band (blue line) at 467 nm. Cr(VI) has three absorption peaks located at 260 nm, 350 nm, and 450 nm (black line), which show quite precise overlap with the excitation and emission bands of *N*,*Cl*-CDs. As such, the detection mechanism is due to the fluorescence IFE with Cr(VI), on one hand shielding the excitation light for *N*,*Cl*-CDs, and on the other hand absorbing the emission light from *N*,*Cl*-CDs. This kind of IFE-based fluorescence quenching sensor has also been designed by other researchers for the detection of Cr(VI) [21,30,42]. In comparison with the fluorescence resonance energy transfer (RET) method, the IFE process has no covalent bonds between an absorber–fluorophore pair [43]. The IFE-based sensing method could provide enhanced sensitivity and fast response as the changes in the absorbance of *N*,*Cl*-CDs could lead to an exponential decrease of the fluorescence intensity [30]. In our work, the IFE process between *N*,*Cl*-CDs and Cr(VI) could occur in a fast and sensitive way with a response time of ≤1 min. 

The quenching mechanism is usually categorized as static quenching and dynamic quenching. To further explore the quenching mechanism behind the Cr(VI) and *N*,*Cl*-CDs reaction, the fluorescence lifetime of *N*,*Cl*-CDs in the absence and in the presence of Cr(VI), which is a critical parameter to estimate static or dynamic quenching, was studied and the results are shown in Figure 6B. The fluorescent decay curves are well fitted according to a double-exponential function (3) [28] and the relevant data are summarized in Table S2.

*I_(t)_* = *A_1_* exp(−*t*/*τ_1_*) + *A_2_* exp(−*t*/*τ_2_*) (3)

where *τ_1_* and *τ_2_* are the time constants of the two radiative decay channels; *A_1_* and *A_2_* are the corresponding amplitudes. In the absence of Cr(VI) (black line, Figure 6B), the average lifetime of *N*,*Cl*-CDs is 8.67 ns (χ^2^ = 1.153) with the lifetimes of τ_1_ = 4.43 ns (32.86%) and τ_2_ = 10.75 ns (67.14%). In the presence of 10 µM Cr(VI) (red line, Figure 6B), the average fluorescence lifetime of the *N*,*Cl*-CDs is 7.73 ns (χ^2^ = 1.102) with lifetimes of τ_1_ = 2.92 ns (21.91%) and τ_2_ = 9.08 ns (78.09%). The results show that the lifetime of *N*,*Cl*-CDs decreases when Cr(VI) is introduced, indicating that fluorescence sensing belongs to the dynamic quenching process [9]. Collectively, the quenching mechanism between the interaction of *N*,*Cl*-CDs and Cr(VI) is a combination of dynamic quenching and IFE.

### 3.7. Real Samples Analysis 

The feasibility of the proposed fluorescence method for the detection of Cr(VI) in real samples was evaluated with tap water, rain water, and river water samples. A standard addition method was used to minimize the possible matrix effect of coexisting species. For the pretreatment, the tap water sample was used as received while the rain water and river water samples were filtered through 0.45 µm membrane before use. The pretreated water samples were spiked with Cr(VI) at four concentration levels (5, 10, 20 and 30 µM) and then analyzed using the proposed fluorescence method. The results are summarized in Table 1. The experimental results show that no detectable Cr(VI) was found in all the water samples. The recovery of Cr(VI) varies from 97.01% to 103.89% with relative standard deviations (RSDs) of less than 0.82%, suggesting the applicability of the proposed fluorescent method for real water sample analysis. The proposed fluorescent method was compared with the previously reported CDs-based fluorescence methods for the recognition of Cr(VI), and the results are listed in Table 2. The fluorescence method proposed here possesses comparable linear range and relatively low LOD. Even though in some cases, some of the previously reported methods [28,29,30,31] have better sensitivity than our proposed fluorescence method, the reported fabrication methods for CDs usually require an external heat source and time-consuming heating process to assist the carbonization of the carbon source [29,30,31], and some of the methods were only performed in the preliminary test stage while their capability for real sample analyses were unknown [28,29]. In comparison with the other reported methods, our proposed *N*,*Cl*-CDs-based fluorescence method stands out in the use of a simple, ultrafast, and energy-saving synthetic route to fabricate CDs possessing the capability for Cr (VI) detection in real samples.

### 3.8. Cytotoxicity of the N,Cl-CDs

The cytotoxicity of *N*,*Cl*-CDs was assessed using human cervical cancer SiHa cells. The viability of SiHa cells incubated with various concentrations of *N*,*Cl*-CDs (0‒800 mg/mL) was measured through MTT assay. As shown in Figure S3, all the cell viabilities are estimated to be higher than 93% after culturing with *N*,*Cl*-CDs in the concentration range of 0‒800 mg/mL. This indicates the low cytotoxicity and good biocompatibility of *N*,*Cl*-CDs, suggesting that the *N*,*Cl*-CDs could be potentially used for the detection of Cr(VI) in living cells.

## 4. Conclusions

A simple, ultrafast, and energy-efficient approach was for the first time presented to fabricate highly fluorescent *N*,*Cl*-CDs by simply mixing glucose, concentrated HCl, and EDA with the neutralization reaction as the heat source. The as-synthesized *N*,*Cl*-CDs are functionalized with hydroxyl, carboxylic, amine, and alkyl chloride functionalities. As such, the *N*,*Cl*-CDs have high hydrophilicity and water dispersity. An *N*,*Cl*-CDs-based fluorescence method with high selectivity and sensitivity was constructed for Cr(VI) detection via a synergistic effect of dynamic quenching combined with IFE. A good linear quenching was observed in a concentration range of 3–40 µM with an LOD of 0.28 µM (14.6 µg/L). The proposed fluorescence method was used for the recognition of Cr(VI) in different water samples with satisfactory results, demonstrating its promising usage in real sample analysis.

## Figures and Tables

**Table 1:** Recoveries for the detection of Cr(VI) in water samples (*n* = 5).

Sample	Cr(VI) (µM)	Added (µM)	Found (µM)	Recovery (%)	RSD (%)
Tap water	N.D.	5	4.92	98.38	0.41
	N.D.	10	9.94	99.36	0.17
	N.D.	20	20.77	103.89	0.55
	N.D.	30	30.86	102.88	0.54
Rain water	N.D.	5	4.86	97.19	0.12
	N.D.	10	9.81	98.11	0.25
	N.D.	20	19.40	97.01	0.73
	N.D.	30	30.06	100.20	0.82
River water Sample A	N.D.	5	4.92	98.31	0.28
	N.D.	10	9.82	98.15	0.15
	N.D.	20	20.29	101.45	0.29
	N.D.	30	29.32	97.72	0.57
River water Sample B	N.D.	5	4.93	98.57	0.16
	N.D.	10	9.95	99.55	0.29
	N.D.	20	19.66	98.31	0.35
	N.D.	30	30.18	100.59	0.38

N.D.: Not detected in the sample.

**Table 2:** Comparison of CDs-based fluorescence methods for the detection of Cr(VI).

Methods	Fabrication Method	Linear Range	LOD	Sample	Ref.
This work	Neutralization heat; 2 min	3‒40 µM	0.28 µM	Water	
CDs	Hydrothermal; 200 °C, 4 h	1.6‒50 µM	1.6 µM	Water	[42]
Chitosan-CDs	Hydrothermal; 180 °C, 13 h	0‒600 µM	13.8 µM (0.72 mg/L)	Water; soil	[44]
Co(II)-doped CDs	Hydrothermal; 180 °C, 4 h	5‒125 µM	1.17 µM	Water; fish	[19]
N,S-doped CDs	Hydrothermal; 160 °C, 6 h	1‒80 µM	0.86 µM	NA	[45]
P-doped CDs	Microwave Heating; NA	1‒400 µM	0.24 µM	Water	[46]
P,N-doped CDs	Neutralization heat; NA	1.5‒30 µM	0.023 µM	NA	[28]
N,B-doped CDs	Hydrothermal; 180 °C, 4 h	1.39–260 µM	0.28 µM	NA	[47]
N-doped CDs	Hydrothermal; 220 °C, 4 h	0‒50 µM	0.01 µM	NA	[29]
N-doped CDs	Calcination; 400 °C, 20 min	0.5‒160 µM	0.15 µM	Water	[30]
N-doped CDs	Pyrolysis; 170 °C, 30 min	0.01‒50 µM	NA	NA	[21]
N-doped CDs	Pyrolysis; 200–240 °C, 2 h	0.2–2 and 2–40 μM	0.018 and 0.25 μM	Water	[31]

CDs: carbon dots; Chitosan-CDs: chitosan-modified CDs; NA: not mentioned in publication.

## Supplementary Materials

The following are available online at http://www.mdpi.com/1424-8220/18/10/3416/s1. Experimental: Characterization of the as-synthesized N,Cl-CDs; Quantum yield (QY) measurements. Table S1. Elemental analysis of the as-fabricated *N*,*Cl*-CDs: (A) elemental content and (B) relative number of atom in *N*,*Cl*-CDs. Figure S1. FTIR spectrum of *N*,*Cl*-CDs. Figure S2. Plots of integrated PL intensity against the absorbance of (A) *N*,*Cl*-CDs and (B) quinine sulfate at λ_ex_/emission wavelength (λ_em_) of 381/468 nm. Table S2. Double-exponential fitting of *N*,*Cl*-CDs and *N*,*Cl*-CDs/Cr(VI) decay curves. Figure S3. Cell viability test of *N*,*Cl*-CDs on SiHa cells. The values represent percentage cell viability (mean% ± SD, *n* = 6).
