# Supplementary material for: Ultrafast and Energy-saving Synthesis of Nitrogen and Chlorine Co-doped Carbon Nanodots via Neutralization Heat for Selective Detection of Cr(VI) in Aqueous Phase
Source: Sensors (Basel). 2018 Oct 11;18(10):3416. doi: 10.3390/s18103416 (PMC6210692; doi:10.3390/s18103416)
Supplement: Supplementary file 1 [file sensors-18-03416-s001.pdf]

Supplementary Material

Ultrafast and Energy-saving Synthesis of Nitrogen and Chlorine Co-doped Carbon Nanodots via Neutralization Heat for Selective Detection of Cr(VI) in Aqueous Phase

Qin Hu [1,2], Tao Li [3], Lu Gao [1,2], Xiaojuan Gong [4], Shengqi Rao [1,2], Weiming Fang [1,2], Ruixia Gu [1,2] and Zhenquan Yang [1,2,*]

[1] College of Food Science and Engineering, Yangzhou University, Jiangsu 225001, China; qinhu1998@outlook.com (Q.H.); gaolu@yzu.edu.cn (L.G.); sqrao@yzu.edu.cn (S.R.); wmfang@yzu.edu.cn (W.F.); rxgu@yzu.edu.cn (R.G.)

[2] Jiangsu Key Laboratory of Dairy Biotechnology and Safety Control, Yangzhou University, Jiangsu 225001, China

[3] Department of Agronomy, Yangzhou University, Jiangsu 225001, China; taoli@yzu.edu.cn

[4] Institute of Environmental Science, and School of Chemistry and Chemical Engineering, Shanxi University, Taiyuan 030006, China; gxj1124@sxu.edu.cn

* Correspondence: yangzq@yzu.edu.cn (Z.Y); Tel.: +86-0514-8978-6037

Experimental

Quantum Yield (QY) Measurements

The quantum yield (Φ_s) of the as-fabricated *N*,*Cl*-CDs sample was determined by a comparative method. The quinine sulfate (Φ_R = 0.54) in 0.10 M H_2SO_4 (refractive index, η = 1.33) was used as the reference to determine the Φ_S of the *N*,*Cl*-CDs sample in ultrapure water (η = 1.33) at different concentrations. A UV-2550 absorption spectrophotometer (Shimadzu Co., Ltd., Tokyo, Japan) was used to record all the absorbances of the solutions at 380 nm. A RF-5301PC fluorescence spectrophotometer (Shimadzu Co., Ltd., Tokyo, Japan) was used to record their PL spectra with an excitation wavelength (λ_{ex}) of 380 nm. The integrated PL intensity was the area under the PL curve in the wavelength range of

400–600 nm. Then a graph of the integrated PL intensity against the absorbance was plotted. The Φ_S of the *N*,*Cl*-CDs sample was calculated using equation (1):

$$\Phi_S = \Phi_R \, (Grad_S \, / \, Grad_R) \, (\eta^2{}_S \, / \, \eta^2{}_R) \quad (1)$$

where Grad is the gradient from the plot of the integrated PL intensity against the absorbance, and η is the refractive index of the solvent. The subscripts S and R denote the sample and reference, respectively. In order to minimize the self-absorption effect, the absorbances in the 10-mm path-length fluorescence cuvette was kept under 0.01 at the λ_{ex} of 380 nm[1–2].

References:

1. Zhu, S.; Meng, Q.; Wang, L.; Zhang, J.; Song, Y.; Jin, H.; Zhang, K.; Sun, H.; Wang, H.; Yang, B., Highly Photoluminescent Carbon Dots for Multicolor Patterning, Sensors, and Bioimaging. *Angewandte Chemie International Edition* **2013,** 52, (14), 3953-3957.
2. Tian, T.; He, Y.; Ge, Y.; Song, G., One-pot synthesis of boron and nitrogen co-doped carbon dots as the fluorescence probe for dopamine based on the redox reaction between Cr(VI) and dopamine. *Sensors and Actuators B: Chemical* **2017,** 240, 1265-1271.

Table S1. Elemental analysis of the as-fabricated *N*,*Cl*-CDs: (A) elemental content and (B) relative number of atom in *N*,*Cl*-CDs.

**Sample**	**Elemental content**				
	C	H	N	Cl	O (calculated)
N,*Cl*-CDs	25.88%	9.40%	25.69%	23.29%	15.74%

**Sample**	**Relative number of atom**					**Empirical formula**
	C	H	N	Cl	O	
N,*Cl*-CDs	6	28	6	2	3	$C_6H_{28}N_6Cl_2O_3$

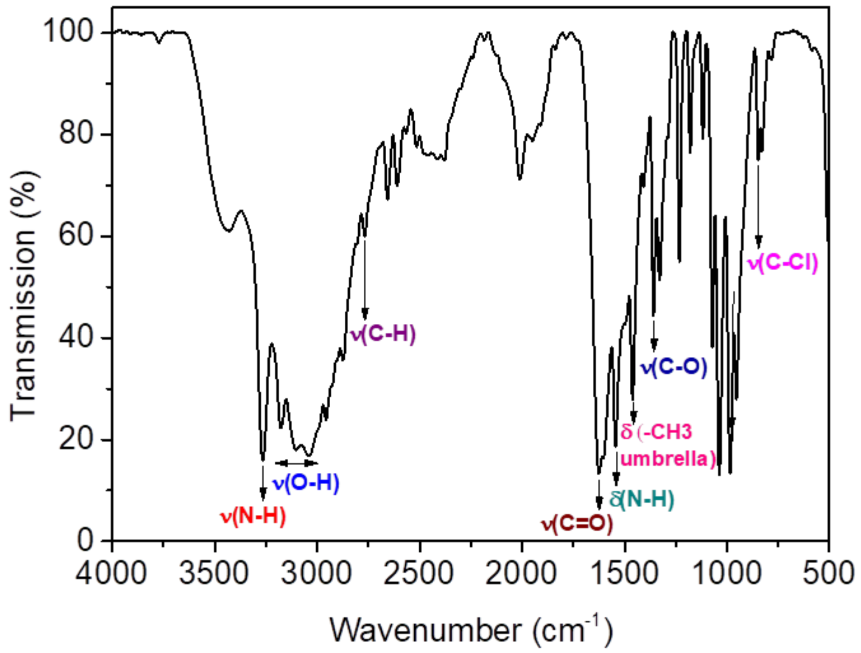

Figure S1. FTIR spectrum of *N*,*Cl*-CDs.

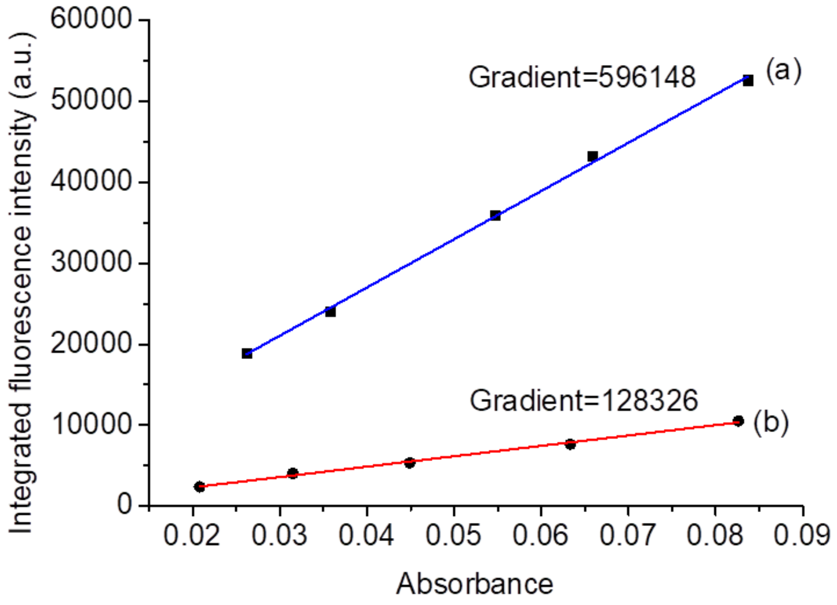

Figure S2. Plots of integrated PL intensity against the absorbance of (A) *N*,*Cl*-CDs and (B) quinine sulfate at λ_{ex}/emission wavelength (λ_{em}) of 381/468 nm.

Table S2. Double-exponential fitting of *N,Cl*-CDs and *N,Cl*-CDs/Cr(VI) decay curves.

Sample name	*N,Cl*-CDs	*N,Cl*-CDs/Cr(VI)
τ_1(ns)/A_1(%)	4.43/32.86	2.92/21.91
τ_2(ns)/A_2(%)	10.75/67.14	9.08/78.09
Average τ(ns)	8.67	7.73

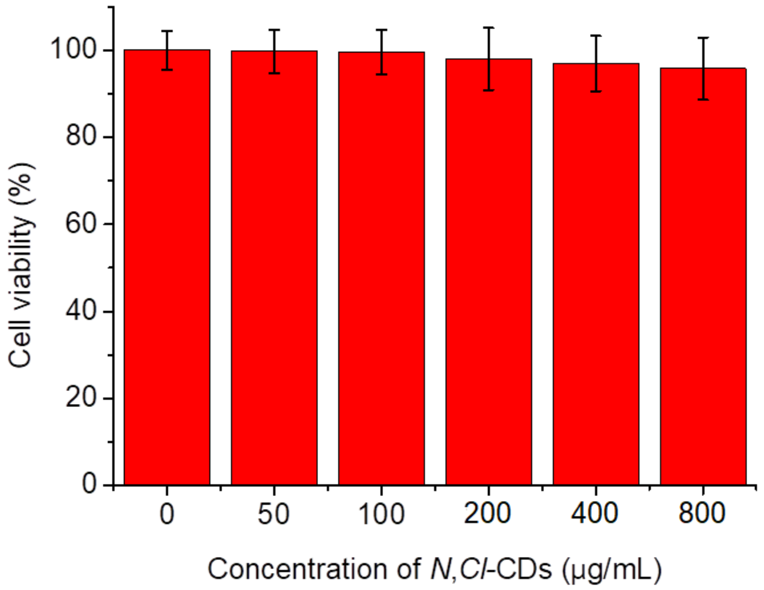

Figure S3. Cell viability test of *N,Cl*-CDs on SiHa cells. The values represent percentage cell viability (mean% ± SD, n=6).
